# Incidence and clearance of anal high-risk Human Papillomavirus infection and their risk factors in men who have sex with men living with HIV

**DOI:** 10.1038/s41598-021-03913-5

**Published:** 2022-01-07

**Authors:** Maria Gabriella Donà, Massimo Giuliani, Francesca Rollo, Maria Fenicia Vescio, Maria Benevolo, Amalia Giglio, Eugenia Giuliani, Aldo Morrone, Alessandra Latini

**Affiliations:** 1 STI/HIV Unit, San Gallicano Dermatological Institute IRCCS, Via Elio Chianesi 53, 00144 Rome, Italy; 2 Pathology Department, Regina Elena National Cancer Institute IRCCS, Via Elio Chianesi 53, 00144 Rome, Italy; 3 Infectious, Parasitic and Immunomediated Diseases Department, Istituto Superiore di Sanità, Viale Regina Elena 299, 00161 Rome, Italy; 4 Microbiology and Clinical Pathology Department, San Gallicano Dermatological Institute IRCCS, Via Elio Chianesi 53, 00144 Rome, Italy; 5 Scientific Direction, San Gallicano Dermatological Institute IRCCS, Via Elio Chianesi 53, 00144 Rome, Italy

**Keywords:** Risk factors, Human papilloma virus

## Abstract

HIV-infected men who have sex with men (MSM) display the highest prevalence of anal infection by high-risk Human Papillomaviruses (hrHPVs) and incidence of anal carcinoma. Anal specimens were genotyped by the Linear Array. Incidence and clearance of anal infection by hrHPVs, hrHPVs other than HPV16, low-risk HPVs, and four individual types (6,11,16,18) were estimated using a two-state Markov model. Determinants for incidence and clearance were assessed by logistic regression. Overall, 204 individuals were included (median age 42 years, IQR = 34–49). For hrHPVs, incidence and clearance rates were 36.1 × 1000 person-months (p-m) (95% CI 23.3–56.5) and 15.6 × 1000 p-m (95% CI 10.7–23.3), respectively. HPV16 showed a higher incidence than HPV18 (10.2 *vs.* 7.2 × 1000 p-m). Its clearance was more than twofold lower than that of HPV18 (30.1 *vs.* 78.2 × 1000 p-m). MSM receiving cART displayed a 68% to 88% decrease in risk of acquiring hrHPVs, hrHPVs other than HPV16, HPV16, and HPV18 (adjusted Hazard Ratio [aHR] 0.13, 95% CI 0.02–0.67; aHR 0.22, 95% CI 0.06–0.78; aHR 0.32, 95% CI 0.12–0.90; aHR 0.12, 95% CI 0.04–0.31, respectively) than patients not treated. A nadir CD4 + count < 200 cells/mm^3^ significantly reduced the clearance of hrHPVs other than HPV16 (aHR 0.39, 95% CI 0.17–0.90). cART use reduces the risk of acquiring anal infection by hrHPVs.

## Introduction

High-risk Human Papillomaviruses (HPVs) are the main causative agents of anal squamous cell carcinoma (hereafter anal cancer), accounting for virtually all cases worldwide^1^. Men who have sex with men (MSM) co-infected with HIV-1 show the highest incidence of anal cancer and are therefore considered as one of the priority target populations for anal cancer prevention^2^.

HPV16 represents the genotype with the highest prevalence in anal infections, irrespective of the presence and severity of anal lesions^3^. In addition, HPV16 causes the majority of anal cancers, regardless the population^4,5^. Interestingly, HPV16 causes a smaller proportion of cases in HIV-positive compared to HIV-negative men^5^. The proportion of cases attributable to other genotypes, including HPV18, is in fact higher in the former than the latter group of subjects^5^.

Numerous cross-sectional studies allowed us to gather comprehensive data on anal HPV prevalence, especially in HIV-infected MSM. Differently, the natural history of the infection in this group, and particularly the risk factors for incidence and clearance, deserve further investigation^6–20^. In this regard, conflicting findings have been obtained, especially about the role of HIV-related factors. In particular, combined antiretroviral therapy (cART) impact on anal HPV infection still needs to be dissected. This knowledge would be of great value especially now that the therapy has so remarkably improved the health status of HIV patients and that new drugs and combinations are available. Since 2015, HIV treatment guidelines, prompted by the evidences of the START study^21^, have recommended to initiate cART at diagnosis regardless CD4 + cell count^22,23^. Therefore, further research is needed to clarify whether and how cART affects the incidence and clearance of anal HPV infection.

In this study, we evaluated a cohort of HIV-infected MSM with the aim of assessing the incidence and clearance rates of anal infection by high-risk HPVs, high-risk HPVs other than HPV16, HPVs 16 and 18. Incidence and clearance were also estimated for low-risk HPVs and their most representative types, i.e., HPVs 6 and 11. In addition, we investigated the risk factors related with incidence and clearance of high-risk HPVs, high-risk HPVs other than HPV16, HPV16 and 18.

## Methods

### Study cohort

Study participants were recruited among all the MSM (defined as men who reported at least one sexual intercourse with another man in the preceding 6 months) attending the STI/HIV Unit of the San Gallicano Dermatologic Institute (Rome, Italy). Those participating in the Surveillance Program of Anal Intraepithelial Neoplasia (SAIN) project^24^ between November 2009 and December 2019 were included in the present longitudinal study based on the following inclusion criteria: (i) age ≥ 18 years; (ii) infection with HIV; (iii) attendee of our centre for the clinical management of HIV infection and antiretroviral therapy administration. The last criterion was motivated by the need to maximize retention in follow-up. In fact, our patients were more likely to be retained in follow-up compared to patients attending our centre from other urban HIV facilities for an anal PAP test or a dermatovenereological consultation. The exclusion criteria were as follows: (i) previous prophylactic HPV vaccination; (ii) presence of ano-genital warts ascertained by visual inspection of external genitalia and perianal area conducted by an expert dermatovenereologist (patients with ano-genital warts were treated and thus excluded from the study, given that the natural history of HPV infection is modified by the treatment); (iii) history of anal cancer. In addition, all HIV-infected MSM had an anal Pap test but having an abnormal cytological report did not represent an exclusion criterion, as in other studies^7,14,15,17^. At baseline, a structured questionnaire was administered to each participant during a face-to-face interview to collect socio-demographic characteristics as well as lifetime and recent (previous 6 months) behavioural data. HIV-related data were retrieved from the medical records. A written informed consent was obtained from each participant. The study was conducted in accordance with the ethical standards of the Helsinki Declaration and was approved by the local Ethics Committee Sezione I.F.O. (Istituti Regina Elena e San Gallicano) – Fondazione Bietti (Prot. CE/564/11).

### Anal sampling

Anal samples were obtained at baseline and each 6-month follow-up visit. They were collected by an expert clinician using a Dacron swab that was inserted into the anal canal and rotated for 60 s. The epithelial cells were dislodged by vigorously agitating the swab in 20 ml of PreservCyt (Hologic, Pomezia, Italy).

### HPV DNA detection and genotyping

HPV DNA was detected and genotyped using the Linear Array HPV Genotyping Test (Roche, Milan, Italy) following the manufacturer’s instructions. Briefly, 250 μl of the PreservCyt sample was used for extraction of total nucleic acids. A region in L1 open reading frame was targeted for amplification by polymerase chain reaction. Hybridization of the denatured amplicons on a strip-immobilized array containing probes for 37 HPV types and subsequent detection were carried out in the Profiblot T48 (Tecan, Männedorf, Switzerland). Whenever the amplification of the β-globin controls and/or at least one specific HPV hybridization band were observed, the HPV test was considered as valid.

### Statistical analysis

Descriptive statistical techniques were used to provide a summarized description of the study population. Participants who provided at least two anal samples for HPV detection were included in the analysis. Incidence and clearance rates were jointly evaluated by using a two-state Markov model for interval-censored observations, as previously detailed^25^. We opted for the use of a two-state Markov model to account for unobserved events between visits and obtain more reliable estimates of the longitudinal measures. In fact, there may be several unobserved incident and cleared infections that occur between samplings. Unlike the Markov model, these are not taken into account by standard person-time analyses, which thus generate downward-biased estimates of the incidence and clearance rates. Geskus and collaborators showed that incidence and clearance rates of anal HPV infection estimated with standard person-time analyses are 20 to over 50% lower compared to those obtained using the two-state Markov model^14^. In our model, individuals could be in one of two possible states, i.e., they could be HPV-DNA-negative (state 0) or HPV-DNA-positive (state 1). At any time point, the individuals could move from one state to the other. Incidence, defined as the rate of transition from a negative to a positive state (from state 0 to state 1), was measured whenever HPV genotype(s) not previously present were detected. Clearance, defined as the rate of transition from a positive to a negative state (from state 1 to state 0), was measured whenever the HPV genotype(s) already present were no longer detected. Standard errors of the rates were calculated by the delta method. Multiple infections detected or cleared during a visit were treated as a single event. The model assumes that future transitions only depend on the current state and that the sojourn time in each state has an exponential distribution. Transition probabilities were calculated analytically from the transition intensity matrix by using the methods described by Cox and Miller^26^. The Markov model was fitted in R (http://www.r-project.org, R Core Team 2021) using the msm package^27^.

Incidence and clearance rates were estimated for: (i) high-risk HPVs: at least 1 high-risk genotype (16, 18, 31, 33, 35, 39, 45, 51, 52, 56, 58, 59, 66 and 68), regardless of the presence of low-risk HPVs; (ii) high-risk HPVs other than HPV16 (18, 31, 33, 35, 39, 45, 51, 52, 56, 58, 59, 66 and 68); (iii) low-risk HPVs: at least 1 low-risk genotype (6, 11, 40, 42, 54, 61, 72, 81, CP6108); (iv) HPVs 6, 11, 16, 18 as individual genotypes.

The risk factors for incidence and clearance were investigated for the subset of participants who agreed to undergo the face-to-face interview. Covariates were fitted to transition intensities using a proportional intensities model. For the purpose of analysis, the following continuous variables were used as categorical variables: (i) age (cut-point at the median); (ii) lifetime number of partners (cut-points at the tertiles); (iii) recent (during the previous 6 months) number of partners (cut-points at the tertiles).

Covariates were entered univariately in transition models. Those found to be significantly associated with incidence and/or clearance were included in the multivariate model. To select the appropriate set of predictors to be included in the final model, a backward stepwise procedure was performed using *p* > 0.05 of the likelihood ratio test for exclusion. Time-dependent covariates were assumed to be piecewise-constant. Transition probabilities and boot-strapped confidence intervals were predicted at 1 to 48 months. Statistical analyses were conducted using R version 4.0.3 (http://www.r-project.org, R Core Team 2021).

## Results

### Study cohort

During the study period, 548 HIV-infected MSM attended our unit. Of these, 287 (52.4%) were enrolled in the longitudinal study (i.e., they agreed to participate, met the inclusion criteria and were not excluded based on the exclusion criteria). Of these, 204 attended at least two visits (loss to follow-up: 28.9%) and were thus included in the analysis. The participants did not differ significantly from those excluded from the longitudinal study regarding age, lifetime and recent number of partners (*p* value from t-test > 0.05 for all the comparisons, data not shown), and also regarding partnership, history of sexually transmitted infections, smoking status and HIV-related parameters (i.e., HIV-1 RNA, nadir and current CD4 + T-cell counts) (Fisher exact *p* value > 0.05 for all the comparisons, data not shown). A significant difference was only found with respect to receptive anal intercourse: the proportion of those consistently using condom during receptive anal sex was higher among the study participants (p = 0.01, data not shown).

The median age of the participants at study entry was 42 years (IQR 34–49). Of the 204 individuals, 187 agreed to the interview (91.7%). Among the respondents, the median number of partners, lifetime and recently, were 60 (IQR 23–175) and 3 (IQR 1–6), respectively. Other behavioral characteristics of the study subjects are shown in Table 1. The large majority of the participants (155/187, 82.9%) reported ever engaging in receptive anal sex. Around a third of these subjects (55/155, 35.5%) referred inconsistent condom use during this sexual practice.

**Table 1:** Behavioral characteristics of the study cohort composed by 204 HIV-infected MSM participating in the Surveillance Program of Anal Intraepithelial Neoplasia (SAIN project) of an STI/HIV Unit in Rome (Italy).

	HIV-infected MSM N = 204
	n (%)
**No. lifetime partners**	
< 35	63 (30.9)
35–100	44 (21.6)
> 100	80 (39.2)
Unknown	17 (8.3)
**No. recent partners**	
0	17 (8.3)
1–5	101 (49.6)
> 5	69 (33.8)
Unknown	17 (8.3)
**Occasional partners in recent sex**	
No	63 (30.9)
Yes	124 (60.8)
Unknown	17 (8.3)
**RAI**	
No	32 (15.7)
Yes, with condom	100 (49.0)
Yes, without condom	55 (27.0)
Unknown	17 (8.3)
**STI history**	
No	54 (26.5)
Yes	133 (65.2)
Unknown	17 (8.3)
**Smoking status**	
Never	99 (48.6)
Former	18 (8.8)
Current	70 (34.3)
Unknown	17 (8.3)

*RAI* receptive anal intercourse, *STI* sexually transmitted infection (ano-genital warts, syphilis, gonorrhea, chlamydia, genital herpes).

Median time since HIV diagnosis was 4.4 years (IQR 2.1–9.8). Nadir and current counts of CD4 + T-cells (CD4 + count) had a median of 304 (IQR 208–400) and 577 cells/mm^3^ (IQR 426–750), respectively. One-hundred eighty-five patients/204 (90.7%) were already on cART at enrolment (median duration: 4.0 years, IQR 1.4–9.2) and most of them (160/185, 86.5%) were virologically suppressed (HIV-RNA < 40 copies/ml).

The 204 participants provided a total of 589 anal samples (median number: 3, range: 2–8). The median time between samplings was 7.9 months (IQR 5.2–13.5). The median duration of follow-up was 38 months (IQR 19–57). None of the study participants underwent prophylactic HPV vaccination during the follow-up period.

Baseline anal prevalence of high-risk HPVs, high-risk HPVs other than HPV16, HPV16 and HPV18 were 77.5% (95% CI 71.1–83.0), 74.5% (95% CI 68.0–80.3), 28.9% (95% CI 22.8–35.7), and 9.8% (95% CI 6.1–14.7), respectively. Baseline anal prevalence of low-risk HPVs, HPV6, HPV11 and were 74.5% (95% CI 68.1–80.0), 24.5% (95% CI 19.1–30.8) and 7.8% (95% CI 4.9–12.4), respectively.

### Incidence and clearance rates of anal HPV infection

Incidence and clearance rates for the HPV groups and individual HPVs included in the analysis, estimated for the entire cohort of 204 participants, are shown in Table 2. Incidence rates of 36.1 × 1000 p-m (95% CI 23.3–56.5) and 32.3 × 1000 p-m (95% CI 21.1–49.1) were calculated for high-risk and low-risk HPVs, respectively. The corresponding clearance rates were 15.6 × 1000 p-m (95% CI 10.7–23.3) and 23.1 × 1000 p-m (95% CI 16.0–33.2). Among the four individual HPVs, i.e., 6, 11, 16 and 18, HPV16 showed the highest incidence rate (10.2 × 1000 p-m, 95% CI 6.9–15.1), followed by HPV18 (7.2 × 1000 p-m, 95% CI 4.0–12.8). Regarding the clearance rate, this was the highest for HPV18 (78.2 × 1000 p-m, 95% CI 47.2–133.2), being the rates for the other three genotypes approximately 2 to 3 times lower than that of HPV18.

**Table 2:** Incidence and clearance rates for the different HPV groups and individual genotypes among the study cohort composed of 204 HIV-infected MSM participating in the Surveillance Program of Anal Intraepithelial Neoplasia (SAIN project) of an STI/HIV Unit in Rome (Italy).

Anal HPV infection	Incidence rate	Clearance rate
	× 1000 p-m (95% CI)	× 1000 p-m (95% CI)
High-risk HPVs	36.1 (23.3–56.5)	15.6 (10.7–23.3)
High-risk HPVs other than 16	30.3 (20.1–45.3)	19.9 (14.3–27.8)
HPV16	10.2 (6.9–15.1)	30.1 (21.3–41.4)
HPV18	7.2 (4.0–12.8)	78.2 (47.2–133.2)
Low-risk HPVs	32.3 (21.1–49.1)	23.1 (16.0–33.2)
HPV6	3.5 (2.0–6.2)	24.4 (16.3–37.4)
HPV11	2.2 (1.1–3.9)	27.5 (14.6–49.3)

**Figure 1:**
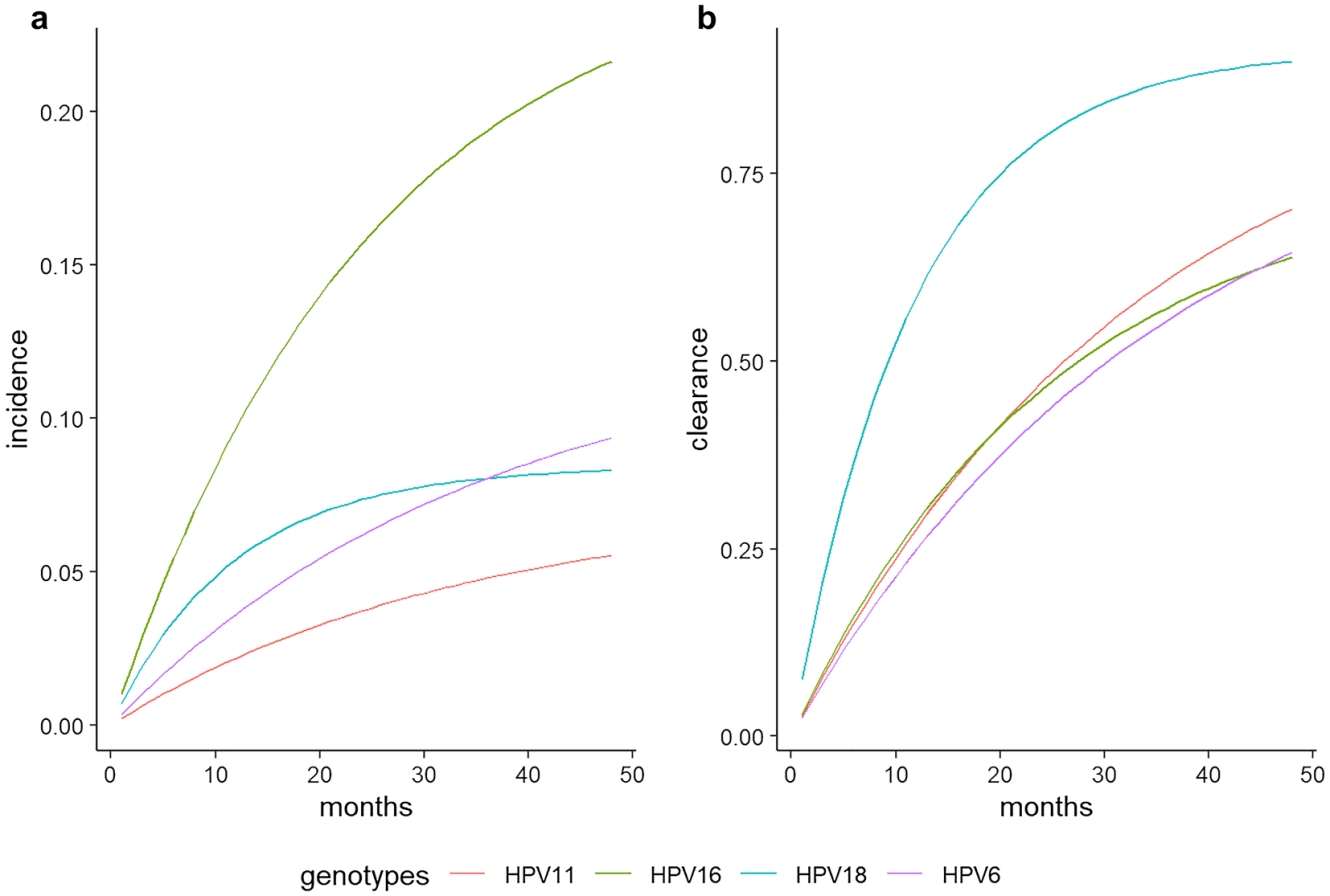
Plots of the transition probabilities for (**a**) the acquisition and (**b**) clearance of the high-risk HPVs 16 and 18 and low-risk HPVs 6 and 11 from month 1 to 48, obtained with a two-state Markov model, among 204 HIV-infected MSM enrolled in the Surveillance Program of Anal Intraepithelial Neoplasia (SAIN) project of an STI/HIV Unit in Rome (Italy).

Figure 1 shows the transition probabilities estimated for HPV 6, 11, 16 and 18 from month 1 to 48. It is predicted that at 24 months from enrolment, 15.2% (95% CI 10.7–20.8) of the subjects will have acquired an HPV16 infection and 8.4% (95% CI 5.2–11.6) an HPV18 infection, whereas the corresponding predicted probabilities for the low-risk HPVs were around 3–6%. At 24 months, approximately three-quarters of the MSM (76.1%, 95% CI 56.0–89.1) are expected to have cleared HPV18 infection, and around half of the subjects the infection caused by HPV16 (46.0%; 95% CI 34.9–57.6).

### Risk factors for incidence and clearance of anal HPV infection

The results of the univariate and multivariate analyses for the incidence and clearance of anal HPV infection by high-risk HPVs and high-risk HPVs other than HPV16 are shown in Table 3. The respective findings for HPV16 and HPV18 are displayed in Table 4. In the multivariate analysis, cART users showed a significant decrease in the incidence of high-risk HPVs, high-risk HPVs other than HPV16, and the high-risk HPVs analyzed separately, i.e. HPV16 and HPV18. In detail, those who were on cART showed an 87% lower incidence of high-risk HPVs (aHR 0.13, 95% CI 0.02–0.67) and 75% lower incidence of high-risk HPVs other than HPV16 (aHR 0.22, 95% CI 0.06–0.78), compared to the subjects who did not have initiated the therapy (consistently with the recommendations of the treatment guidelines at the time of their enrolment). In those receiving cART, a significant decrease in the acquisition of HPV16 (aHR 0.32, 95% CI 0.12–0.90) and HPV18 (aHR 0.12, 95% CI 0.04–0.31) was also observed.

**Table 3:** Univariate and multivariate analyses for risk factors of incidence and clearance of the anal infection by high-risk HPVs and high-risk HPVs other than HPV16 among 187 HIV-infected MSM participating in the Surveillance Program of Anal Intraepithelial Neoplasia (SAIN) project of an STI/HIV Unit in Rome (Italy).

Anal HPV infection	Univariate				Multivariate			
	Incidence rate		Clearance rate		Incidence rate		Clearance rate	
	HR	(95% CI)	HR	(95% CI)	aHR	(95% CI)	aHR	(95% CI)
**High-risk HPVs**								
Age (> 42 vs. < 42 years)	0.56	(0.22–1.48)	1.02	(0.48–2.20)				
No. lifetime partners (36–100 vs. < 36)	1.67	(0.66–4.22)	0.93	(0.44–1.98)				
No. lifetime partners (> 100 vs. < 36)	1.40	(0.64–3.05)	0.75	(0.38–1.47)				
No. recent partners (1–5 vs. 0)	1.89	(0.33–10.83)	3.63	(0.52–25.37)				
No. recent partners (> 5 vs. 0)	2.24	(0.38–13.05)	3.80	(0.54–26.91)				
Occasional partners in recent sex (yes vs. no)	0.76	(0.37–1.56)	1.16	(0.61–2.20)				
RAI with condom vs. no RAI	1.17	(0.45–3.00)	1.62	(0.54–4.88)				
RAI without condom vs. no RAI	2.30	(0.83–6.36)	1.80	(0.57–5.76)				
Smoking status (current vs. never/former smoker)	0.63	(0.29–1.35)	0.99	(0.54–1.82)				
STI history (yes vs. no)	1.24	(0.59–2.62)	1.35	(0.69–2.66)				
Nadir CD4 + (< 200 vs. ≥ 200 cells/mm^3^)	1.15	(0.42–3.17)	0.62	(0.27–1.44)				
Current CD4 + (< 500 vs. ≥ 500 cells/mm^3^)	0.89	(0.43–1.85)	0.98	(0.53–1.84)				
cART (yes vs. no)	0.13	(0.02–0.67)	3.50	(0.83–14.76)	0.13	(0.02–0.67)		
HIV-1 RNA (undetectable vs. detectable)	0.52	(0.22–1.18)	1.15	(0.56–2.37)				
**High-risk HPVs other than 16**								
Age (> 42 vs. < 42 years)	0.45	(0.17–1.18)	1.22	(0.61–2.43)				
No. lifetime partners (36–100 vs. < 36)	1.15	(0.48–2.76)	1.05	(0.53–2.09)				
No. lifetime partners (> 100 vs. < 36)	1.47	(0.70–3.08)	0.60	(0.32–1.13)				
No. recent partners (1–5 vs. 0)	1.16	(0.30–4.43)	1.74	(0.54–5.63)				
No. recent partners (> 5 vs. 0)	1.65	(0.42–6.43)	1.45	(0.44–4.81)				
Occasional partners in recent sex (yes vs. no)	0.97	(0.50–1.89)	0.89	(0.50–1.59)				
RAI with condom vs. no RAI	1.17	(0.47–2.90)	0.92	(0.41–2.06)				
RAI without condom vs. no RAI	2.10	(0.79–5.60)	1.06	(0.45–2.51)				
Smoking status (current vs. never/former smoker)	0.92	(0.45–1.86)	0.80	(0.46–1.39)				
STI history (yes vs. no)	1.09	(0.54–2.18)	1.21	(0.66–2.22)				
Nadir CD4 + (< 200 vs. ≥ 200 cells/mm^3^)	1.95	(0.75–5.06)	0.43	(0.18–0.99)			0.39	(0.17–0.90)
Current CD4 + (< 500 vs. ≥ 500 cells/mm^3^)	0.89	(0.43–1.84)	0.84	(0.46–1.52)				
cART (yes vs. no)	0.21	(0.06–0.78)	2.40	(0.76–7.60)	0.22	(0.06–0.78)		
HIV-1 RNA (undetectable vs. detectable)	0.77	(0.36–1.65)	0.94	(0.49–1.82)				

*cART* combined antiretroviral therapy, *CI* confidence interval, *HR* hazard ratio, *aHR* adjusted hazard ratio, *RAI* receptive anal intercourse, *STI* sexually transmitted infection.

**Table 4:** Univariate and multivariate analyses for risk factors of incidence and clearance of the anal infection by HPV16 and HPV18 among 187 HIV-infected MSM participating in the Surveillance Program of Anal Intraepithelial Neoplasia (SAIN) project of an STI/HIV Unit in Rome (Italy).

Anal HPV infection	Univariate				Multivariate			
	Incidence rate		Clearance rate		Incidence rate		Clearance rate	
	HR	(95% CI)	HR	(95% CI)	aHR	(95% CI)	aHR	(95% CI)
**HPV16**								
Age (> 42 vs. < 42 years)	0.56	(0.22–1.44)	0.89	(0.39–2.03)				
No. lifetime partners (36–100 vs. < 36)	1.64	(0.63–4.29)	0.39	(0.15–1.02)				
No. lifetime partners (> 100 vs. < 36)	1.46	(0.63–3.38)	0.67	(0.33–1.35)				
No. recent partners (1–5 vs. 0)	0.93	(0.28–3.13)	0.91	(0.30–2.82)				
No. recent partners (> 5 vs. 0)	0.79	(0.22–2.80)	1.10	(0.35–3.43)				
Occasional partners in recent sex (yes vs. no)	0.84	(0.40–1.75)	1.77	(0.83–3.79)				
RAI with condom vs. no RAI	0.48	(0.20–1.17)	0.97	(0.42–2.26)				
RAI without condom vs. no RAI	0.75	(0.29–1.93)	1.04	(0.41–2.63)				
Smoking status (current vs. never/former smoker)	0.58	(0.28–1.19)	0.97	(0.51–1.83)				
STI history (yes vs. no)	1.24	(0.57–2.71)	1.15	(0.55–2.40)				
Nadir CD4 + (< 200 vs. ≥ 200 cells/mm^3^)	1.73	(0.75–3.99)	0.43	(0.14–1.33)				
Current CD4 + (< 500 vs. ≥ 500 cells/mm^3^)	0.90	(0.41–1.99)	1.13	(0.54–2.35)				
cART (yes vs. no)	0.32	(0.12–0.90)	3.15	(0.95–10.37)	0.32	(0.12–0.90)		
HIV-1 RNA (undetectable vs. detectable)	0.50	(0.22–1.15)	1.83	(0.80–4.15)				
**HPV18**								
Age (> 42 vs. < 42 years)	0.82	(0.25–2.66)	1.60	(0.59–4.38)				
No. lifetime partners (36–100 vs. < 36)	1.40	(0.35–5.71)	0.82	(0.26–2.54)				
No. lifetime partners (> 100 vs. < 36)	2.19	(0.70–6.88)	0.78	(0.29–2.10)				
No. recent partners (1–5 vs. 0)	1.60	(0.20–12.90)	0.46	(0.11–1.95)				
No. recent partners (> 5 vs. 0)	2.54	(0.32–20.32)	0.54	(0.14–2.10)				
Occasional partners in recent sex (yes vs. no)	1.06	(0.40–2.79)	1.07	(0.41–2.77)				
RAI with condom vs. no RAI	1.09	(0.30–4.01)	0.85	(0.25–2.82)				
RAI without condom vs. no RAI	1.40	(0.34–5.70)	0.43	(0.12–1.58)				
Smoking status (current vs. never/former smoker)	1.09	(0.41–2.86)	0.95	(0.41–2.19)				
STI history (yes vs. no)	0.87	(0.34–2.22)	0.76	(0.30–1.91)				
Nadir CD4 + (< 200 vs. ≥ 200 cells/mm^3^)	1.24	(0.40–3.82)	0.45	(0.13–1.55)				
Current CD4 + (< 500 vs. ≥ 500 cells/mm^3^)	1.06	(0.39–2.88)	0.46	(0.16–1.30)				
cART (yes vs. no)	0.12	(0.04–0.31)	1.76	(0.65–4.79)	0.12	(0.04–0.31)		
HIV-1 RNA (undetectable vs. detectable)	0.29	(0.11–0.77)	1.79	(0.75–4.26)				

*cART* combined antiretroviral therapy, *CI* confidence interval, *HR* hazard ratio, *aHR* adjusted hazard ratio, *RAI* receptive anal intercourse, *STI* sexually transmitted infection.

Nadir CD4 + count emerged as significantly associated with the clearance of high-risk HPVs other than HPV16, with those with < 200 cells/mm^3^ displaying a 61% decreased clearance of infection (aHR 0.39, 95% CI 0.17–0.90).

## Discussion

We conducted a longitudinal study to gain a deeper understanding of the natural history of anal infection by high-risk HPVs in a cohort of HIV-infected MSM, since they show the highest incidence of anal cancer^2^.

Our estimates are consistent with those of other studies conducted on HIV-infected MSM only to a certain degree^13,14^, but different behavioural and clinical characteristics of the study population, HPV testing methods, definitions of events and data analysis profoundly affect the estimates and likely explain divergence in the results.

In our study, the incidence rate for high-risk HPVs did not significantly differ from that of low-risk HPVs, as indicated by the overlapping confidence intervals. Differently, the incidence rates for HPV16 and 18 exceeded that for low-risk types (i.e., HPV6 and 11). Noteworthy, comparing the estimates for the HIV-infected MSM with those we previously obtained for the HIV-uninfected counterparts, also enrolled within the SAIN study and analysed with the same method for the estimation of the longitudinal measures^25^, we observed that the incidence rates of high-risk and low-risk HPVs were similar for the two groups of MSM. Differently, the HIV-infected MSM were found to have a 2 to 4 times lower clearance of high-risk and low-risk HPVs (both at the group level and at the individual level, i.e., HPV 6 and 11 separately) compared to the HIV-uninfected MSM. A similar pattern of results was also observed for HPV16. In fact, whereas its incidence rate was similar in both groups, its clearance rate in the HIV-infected MSM was approximately half of that estimated for the HIV-uninfected counterparts. Taken together, these findings suggest that acquisition of anal HPV in our cohorts of MSM does not differ substantially with the HIV status. Conversely, HIV-infected MSM seems to be less capable of clearing the infection, irrespective of HPV carcinogenicity, confirming findings by others^6,20^. It can thus be argued that the reason for the higher prevalence of anal HPV infection in our cohort of HIV-infected vs. that of HIV-uninfected MSM resides in a lower clearance more than a higher incidence. This scenario seems to apply well to HPV16 prevalence, which in our cohorts of HIV-uninfected and HIV-infected MSM was 16.1% and 28.9%, respectively, in line with the estimates of a recent meta-analysis that showed how anal HPV16 prevalence for HIV-negative MSM is approximately half of that calculated for HIV-positive MSM (14% vs. 30%)^3^. This ratio fits well with our results regarding HPV16 incidence and clearance in the two cohorts, as outlined above, although the picture might not be that clear, given that other studies estimated for high-risk HPVs and HPV16 a twofold higher incidence in HIV-infected than uninfected MSM^10,13,20^, with a similar clearance^10,13^. A recent meta-analysis also showed a significantly increased risk of acquiring high-risk HPVs and HPV16 in HIV-positive subjects compared to HIV-negative ones, although the study analyzed together men and women, and anal and genital infections^28^. In addition, a recent study on HIV-positive MSM with long-standing HIV infection found that lower HPV16 clearance was confined to those with low nadir CD4 + counts^19^.

Compared to the other individual genotypes analyzed in this study, HPV16 showed the highest incidence rate and its clearance rate was more than twofold lower than that of HPV18. Although in terms of absolute numbers our estimates for HPV16 and 18 clearance rates were approximately twice as high those reported in very recent studies^17,20,29^, our results are perfectly in line in terms of ratio between the clearance rates of the two genotypes. These findings may explain the higher prevalence of HPV16 compared to HPV18 and also its unique carcinogenic potential, thus associated with a slower clearance and a higher persistence capacity. The transition probabilities predicted for these two genotypes further underline their different behaviour. As shown by the plot, the probabilities predicted for HPV18 acquisition consistently remains lower than those for HPV16 over the entire time period. In addition, while the curve for HPV18 seems to flatten after 24 months (suggesting that the proportion of subjects expected to acquire HPV18 reaches a plateau), the curve for HPV16 shows a continuous increase overtime of the proportion of MSM expected to acquire the infection by this genotype.

Our risk factor analysis revealed independent associations with cART and nadir CD4 + count. Compared to the subjects who were not under cART at the time of enrolment, MSM receiving cART displayed a 68–88% lower risk of acquiring anal infections by high-risk HPVs, high-risk HPVs other than HPV16, HPV16, and HPV18. Our observations reinforce the data of a very recent meta-analysis showing that effective cART use is associated with a decreased prevalence of anal high-risk HPV infection^30^. Use of cART for more than 5 years was also shown to be associated with increased clearance of high-risk types in another study^18^. Others did not observe any impact of cART on anal HPV natural history^10,17,20,31^, but findings in this regard may be influenced by type of therapy, and its duration, which in our study cohort had a median of 4 years. cART use for shorter periods may not have the same effects we observed on the anal HPV incidence. It is remarkable that MSM with a nadir CD4 + count < 200 cells/mm^3^ showed a significantly reduced clearance of high-risk HPVs other than HPV16, in agreement with the findings by Poynten et al^19^. These results are in line with the more prominent role of these genotypes in anal HSIL and cancers arising in HIV-infected subjects^5^. Additionally, they suggest that starting with a low CD4 + cells count compromises the capacity of clearing anal HPV infection despite recovery of the immune function due to cART effectiveness. Noteworthy, significantly increased odds of incident anal HSIL + in those with a nadir CD4 + count < 200 cells/mm^3^ recently emerged^32^.

Regarding the limitations of our study, we have to acknowledge the low sample size. The fact that less than half of the HIV-infected MSM attending our centre during the study period were included in the longitudinal study may represent a source of selection bias, although the characteristics of the participants did not differ significantly from those of the non-participants, except for the use of condom during receptive anal sex. This, however, did not show association with the outcomes of interest. It could be also argued that the participants had a risky sexual behaviour (high number of lifetime partners, high rate of occasional partnership, high frequency of STI history), thus our results might not be generalizable to other cohorts of HIV-infected MSM. The elevated proportion of aviremic cART users might further limit the generalizability of our findings to HIV patients with poor HIV control. In addition, covariates used for risk factor analysis were related to the baseline and this did not allow us to take into account possible patient shifts across risk categories. The use of cART as binomial covariate (yes *vs.* no) did not allow us to establish the possible impact of different drugs or regimens on the incidence/clearance of anal HPV. Additionally, more stringent definitions for incidence (two consecutive positive tests) and clearance (two consecutive negative tests) were not used to avoid exclusion of participants with only two samples. Finally, we did not estimate the clearance rates separately for incident and prevalent infections, although it has been shown that clearance is higher for incident than prevalent infections^13,19,33^. Our study has also several strengths, mainly residing in our methodological approach. Using the Markov model we took into account the interval-censored nature of the data. Moreover, estimation of incidence and clearance rates was performed simultaneously, therefore participants were not divided in sub-groups based on their HPV status at baseline. Finally, given the duration of the study, which included a period of time prior to the implementation of the new HIV treatment guidelines, patients who were not under therapy could be included in the study, thus allowing us to have a reference group to evaluate cART impact on anal HPV natural history.

In summary, our study allowed us to gather new insights in the understanding of anal HPV natural history in MSM with HIV infection. The present findings, together with our previous data, suggest that anal infections by low-risk and high-risk HPVs are acquired at a similar rate among HIV-infected and -uninfected MSM, but that they are both cleared more slowly by the former subjects. Importantly, cART use was shown to reduce significantly the risk of acquiring anal high-risk HPV infections, including those caused by HPV16 and HPV18. Additional analyses in larger cohorts, also taking into account duration of therapy and type of regimen, would be useful to shed more light on the effect of cART on the natural history of anal HPV. In the meanwhile, early cART initiation and strategies for sustained adherence should be promoted among MSM also for the potential impact on their risk for high-risk HPV anal infection and, ultimately, anal cancer.
