# Author Correction: Incidence and clearance of anal high-risk Human Papillomavirus infection and their risk factors in men who have sex with men living with HIV

**DOI:** 10.1038/s41598-022-14265-z

**Published:** 2022-06-09

**Authors:** Maria Gabriella Donà, Massimo Giuliani, Francesca Rollo, Maria Fenicia Vescio, Maria Benevolo, Amalia Giglio, Eugenia Giuliani, Aldo Morrone, Alessandra Latini

**Affiliations:** 1 STI/HIV Unit, San Gallicano Dermatological Institute IRCCS, Via Elio Chianesi 53, 00144 Rome, Italy; 2 Pathology Department, Regina Elena National Cancer Institute IRCCS, Via Elio Chianesi 53, 00144 Rome, Italy; 3 Infectious, Parasitic and Immunomediated Diseases Department, Istituto Superiore di Sanità, Viale Regina Elena 299, 00161 Rome, Italy; 4 Microbiology and Clinical Pathology Department, San Gallicano Dermatological Institute IRCCS, Via Elio Chianesi 53, 00144 Rome, Italy; 5 Scientific Direction, San Gallicano Dermatological Institute IRCCS, Via Elio Chianesi 53, 00144 Rome, Italy

Correction to: *Scientific Reports* 10.1038/s41598-021-03913-5, published online 07 January 2022

The Funding section in the original version of this Article was incorrect.

“This work was supported by the Italian Ministry of Health (“5 × 1000”).”

now reads:

“This research was funded by the Italian Ministry of Health (RC 2022).”

The original Article has been corrected.

